# Maternal postpartum deworming and infant milk intake: Secondary outcomes from a trial

**DOI:** 10.1111/mcn.13183

**Published:** 2021-03-17

**Authors:** Layla S. Mofid, Martín Casapía, Antonio Montresor, Elham Rahme, Grace S. Marquis, Jozef Vercruysse, Lindsay H. Allen, Brittany Blouin, Hugo Razuri, Lidsky Pezo, Theresa W. Gyorkos

**Affiliations:** ^1^ Department of Epidemiology, Biostatistics and Occupational Health McGill University Montréal Québec Canada; ^2^ WHO Collaborating Centre for Research and Training in Parasite Epidemiology and Control Montréal Québec Canada; ^3^ Centre for Outcomes Research and Evaluation Research Institute of the McGill University Health Centre Montréal Québec Canada; ^4^ Asociación Civil Selva Amazónica Iquitos Peru; ^5^ Department of Control of Neglected Tropical Diseases World Health Organization Geneva Switzerland; ^6^ Department of Medicine McGill University Montréal Québec Canada; ^7^ School of Human Nutrition McGill University Sainte Anne‐de‐Bellevue Québec Canada; ^8^ Department of Virology, Parasitology and Immunology, Faculty of Veterinary Medicine Ghent University Merelbeke Belgium; ^9^ USDA, ARS Western Human Nutrition Research Center University of California Davis California USA

**Keywords:** albendazole, deuterium oxide, female, helminthiasis, lactation, milk, human, mothers

## Abstract

The World Health Organization recommends deworming to reduce soil‐transmitted helminth (STH)‐attributable morbidity in women of reproductive age, including pregnant and lactating women, to reduce blood loss, iron deficiency anaemia and nutrient malabsorption. This study assessed the impact of maternal postpartum deworming with albendazole approximately 1 day after delivery on infant milk intake among a subset of 216 randomly selected mother–infant pairs recruited into a large trial in Peru. Infant milk intake was measured using the deuterium‐oxide method at 1‐ and 6‐month postpartum. Maternal STH infection was measured at 6‐month postpartum. At 1‐month postpartum, mean intake was 756 ± 16 and 774 ± 18 mL day^−1^ in the albendazole and placebo groups, respectively (mean difference: −18 mL day^−1^; 95% CI: −65, 30). At 6‐month postpartum, mean intake was 903 ± 16 and 908 ± 18 mL day^−1^ in the albendazole and placebo groups, respectively (mean difference: −5 mL day^−1^; 95% CI: −52, 43). There was no statistically significant difference in milk intake between groups at either time point. At 6‐month postpartum, mothers infected with *Trichuris trichiura* had infants with higher milk intakes (adjusted mean difference: 70 mL day^−1^; 95% CI: 20, 120) compared with uninfected mothers. However, there was no statistically significant difference in infant milk intake between mothers who had moderate‐and‐heavy intensity infection compared with the comparison group (mothers with no and light intensity infection). A lower prevalence and intensity of infection, and inclusion of uninfected mothers in both arms of the trial, resulting in effect dilution, may explain the null findings.

**Key messages**

- WHO considers women of reproductive age, including pregnant and lactating women, to be a high‐risk group for STH infections. In 2015, it was estimated that nearly 70 million lactating women required preventive chemotherapy for STH.
- At 1‐ and 6‐month postpartum, we found no statistically significant difference in milk intake between infants of mothers administered albendazole or placebo following delivery.
- At 6‐month postpartum, we found a statistically significant increase in milk intake among infants of mothers infected with *T. trichiura* compared with infants of uninfected mothers.
- We recommend that our results be corroborated in higher STH endemicity regions.

## 1 INTRODUCTION

Adequate nutrition during early infancy, particularly during the first 1,000 days from conception to 2 years of age, is imperative for improving infant health and development outcomes (Bhutta et al., 2008, 2013). It is well established that exclusive breastfeeding is the ideal source of infant nutrition during the first 6 months of life (World Health Organization, 2014) and that it is associated with lower infant infection, morbidity and even mortality (Black et al., 2013; Kramer & Kakuma, 2012). The degree of benefit received by the infant during breastfeeding depends on both milk quality (i.e., nutrient composition of milk) and quantity (i.e., infant milk intake) (Abrams, 1991).

Maternal undernutrition during pregnancy and lactation is a global health concern, especially in low‐ and middle‐income countries where low‐nutrient density diets and concurrent risk of infection are common (Ahmed et al., 2012). Infection with roundworms (*Ascaris lumbricoides*), whipworms (*Trichiura trichiura*) and hookworms (*Necator americanus* and *Ancylostoma duodenale*), commonly referred together as the soil‐transmitted helminths (STHs), is endemic in more than 100 countries worldwide (Pullan et al., 2014) and contributes to undernutrition by causing malabsorption of nutrients, loss of appetite, chronic blood loss and iron deficiency anaemia (Crompton & Nesheim, 2002). Women of reproductive age are a high‐risk group for STH infection, particularly infections with hookworm (Mupfasoni et al., 2018; World Health Organization, 1996, 2017). Milk volume and the concentration of some nutrients (e.g., thiamin, riboflavin, vitamins B‐6 and B‐12, vitamin A and iodine) can be affected by maternal nutritional status (Abrams, 1991; Dror & Allen, 2018), compromising both infant nutritional status and development (Dijkhuizen et al., 2001). However, to date, neither the effect of deworming nor of STH infection on lactation performance (i.e., milk composition or quantity of intake) has been studied. The present study therefore aimed to assess the associations between maternal postpartum deworming and maternal STH infection, and the quantity of infant milk intake, using the state‐of‐the‐art dose‐to‐mother deuterium‐oxide turnover technique (Lopez‐Teros et al., 2017).

## 2 METHODS

### 2.1 Study design

This study was embedded into a larger randomized placebo‐controlled trial that aimed to investigate the effectiveness of integrating single‐dose deworming with albendazole into routine maternal postpartum care on infant and maternal health outcomes in Iquitos, Peru between February 2014 and September 2016. Details about the trial can be found elsewhere (Mofid et al., 2015, 2017). The primary outcome of the trial was mean infant weight gain between birth and 6 months of age, and participants were followed up for a period of 24 months. This paper presents results of the secondary outcome of infant milk intake for a subset of participants of the parent trial.

Women were approached in their third trimester of pregnancy using rosters from local health centres. Pregnant women were eligible to participate in the trial if they met the following inclusion criteria: (a) planned delivery at the Hospital Iquitos ‘Cesar Garayar Garcia’; and (b) intention to reside in the study area for the duration of the study period (24 months). When mothers presented at the hospital for delivery, they were reassessed for eligibility using the following exclusion criteria: (a) stillborn infant; (b) infant with a serious congenital abnormality or medical condition; (c) gestational age < 32 weeks; (d) Apgar score < 4 at 5 min; (e) multiple birth; (f) mother or infant being transferred to another hospital prior to discharge; (g) mother or infant being hospitalized for >3 days; and (h) inability to communicate in Spanish. Following delivery and prior to hospital discharge, 1,010 mothers were randomized to receive either a single‐dose 400‐mg tablet of albendazole or matching placebo. A baseline questionnaire was administered to collect information on socio‐demographic characteristics, and anthropometry was performed on all infants following birth. Investigators, research assistants and participants were blinded to treatment allocation. Administration of the albendazole or placebo tablet was directly observed.

During the informed consent process, the prospective mothers and fathers of newborn infants were also asked about their willingness to participate in a study assessing human milk composition and infant milk intake at 1‐ and 6‐month postdelivery. Of those who agreed to participate, a random subset of participants (*n* = 200) was selected, stratified by infant sex. Mother–infant pairs who participated in the 1‐month assessment were intended to also participate in the 6‐month assessment. If mother–infant pairs who completed the first assessment were not available to complete the second assessment, they were replaced with another pair, selected at random from among those who had consented to participate in the study during recruitment.

### 2.2 Questionnaire administration

Prior to random allocation, research assistants administered a baseline questionnaire to mothers to collect information on socio‐demographics, deworming history during pregnancy and iron supplementation. At 1‐ and 6‐month postpartum, mothers were administered a questionnaire to collect information on current breastfeeding practices (e.g., exclusive breastfeeding and introduction of complementary foods). Questions on breastfeeding practice were adapted from the World Health Organization (WHO) indicators for assessing infant and young child feeding practices (World Health Organization, 2008).

### 2.3 Infant milk intake assessment

Water intake from human milk and other sources was estimated in the infants using the dose‐to‐mother deuterium‐oxide turnover technique (Coward, 1984; International Atomic Energy Agency, 2010) at 1‐ and 6‐month following delivery. At each of the 1‐ and 6‐mo assessments, mother–infant pairs were visited in their homes 6 times over a period of 2 weeks. On Day 1, a nonenriched urine sample of at least 3 mL was collected from mothers and their infants to assess baseline levels of deuterium. Following sample collection, mothers were administered a preweighed 10‐g oral dose of deuterium oxide (99.8% purity), accurate to the nearest 0.001 g. Mothers wore an absorbent bib in case of spill. Following administration of deuterium, research assistants added an additional 40 mL of purified water to the vial that contained the deuterium and asked mothers to ingest the remaining liquid. The date and time of deuterium administration were recorded.

Research assistants returned to participants' homes to collect urine samples from the mother on Days 2, 5, 14 and 15 postingestion and to collect urine samples from the infant on Days 2, 4, 5, 14 and 15 postingestion. Urine samples from mothers were collected in presterilized containers, and urine samples from infants were collected using paediatric collection bags. The date and time of urine collection were recorded. Weight and height of mothers were measured at the 1‐ and 6‐month time points using a portable electronic scale accurate to the nearest 0.1 kg and a stadiometer (Seca 213, Seca Corp., Baltimore, MD, USA) accurate to the nearest 0.1 cm. Weight and recumbent length of infants were measured at the 1‐ and 6‐month time points using a digital balance (Seca 354, Seca Corp., Baltimore, MD, USA), accurate to 0.01 kg and a stadiometer (Seca 417, Seca Corp., Baltimore, MD, USA), accurate to 0.1 cm. Urine samples were packed in separate sealed bags to prevent contamination and transported to the laboratory in a cooler on ice. Samples were refrigerated at 4°C until processing.

Upon arrival at the laboratory, all urine samples were aliquoted into sterile 5‐mL cryovials and stored at −20°C. Following specific instructions for sample collection, all samples were shipped on dry ice to the Instituto de Nutrición y Tecnología de los Alimentos, Universidad de Chile (Santiago, Chile) for laboratory assessment. Isotope ratio mass spectrometry was used to measure the ^2^H enrichment of urine samples. Infant intake of human milk and water from other sources was estimated by fitting deuterium enrichment data to a model for water turnover in mothers and infants, using methods that have been previously described and validated (Coward et al., 1982; Haisma et al., 2003, 2005).

Data collected during home visits (e.g., date and time of sample collection, weight and height/length) were recorded electronically in real time on tablets and reviewed daily for accuracy and completeness. Laboratory management of samples (e.g., labelling and storage) were coordinated using the Laboratory Data Management System (Version 11.1.0.30, Frontier Science, Amherst, NY, USA).

### 2.4 Stool specimen analysis

Stool specimens were collected from mothers at the 6‐month time point for the determination of STH prevalence and intensity. Specimens were transferred to the laboratory of the local research facility and processed within 24 h of collection using the Kato–Katz technique (World Health Organization, 1998). One microscope slide per specimen was quantitatively examined for helminth ova by trained technologists. STH prevalence was based on the identification of at least one STH helminth egg during microscopic examination. STH intensity, based on parasite‐specific egg counts, was categorized according to WHO cut‐offs for light, moderate and heavy intensity infection (World Health Organization, 2002).

### 2.5 Power calculations

The study sample size of 200 mother–infant pairs had 80% power to detect a 51‐mL difference in infant milk intake between intervention groups, using a two‐sided Student's *t* test, an alpha value of .05, a common standard deviation of 130 mL (Albernaz et al., 2003; Haisma et al., 2003; International Atomic Energy Agency, 2010; Limon‐Miro et al., 2017) and an approximate 1:1 ratio of participants allocated to the experimental and control groups. Previous published evidence from Mexico and Brazil suggests that a mean infant milk intake difference of approximately 100 mL would be biologically meaningful (Albernaz et al., 2003; Limon‐Miro et al., 2017). Power calculations were performed using PS Power and Sample Size Calculations Version 3.0 (Copyright © 1997 by Dupont and Plummer).

### 2.6 Statistical analysis

Baseline characteristics between those lost to follow‐up and their replacements were compared using a chi‐square test for categorical variables and Student's *t* test for continuous variables. The impact of maternal postpartum deworming on infant milk intake was compared using the intention‐to‐treat (ITT) principle in which participants are analysed according to the groups to which they were randomized.

Crude differences in infant milk intake were compared between groups using Student's *t* test. Multivariable linear regression models were used to evaluate the impact of maternal postpartum deworming on infant water intake with adjustment for covariables that had been determined to be important a priori from a review of the literature. These variables included maternal age, education level, socio‐economic status (SES) index, gestational age and infant sex. The SES index was created by principal components analysis using the following baseline variables: ownership of a radio and television, type of cooking fuel, type of flooring, water connection to the house and wired electricity in the house, as described previously (Filmer & Pritchett, 2001; Gyorkos et al., 2013; Joseph et al., 2014). The SES index was divided into quartiles for inclusion in multivariable models. Sensitivity analyses, including complete‐case and per‐protocol analyses, were conducted to test the robustness of study findings. Subgroup analysis was also performed among those mothers who reported exclusive breastfeeding.

An exploratory analysis of the associations between infant milk intake and maternal STH prevalence and intensity, both measured at the 6‐month time point, was evaluated using multivariable linear regression with adjustment for possible confounders. A factor was considered as a possible confounder if its association with milk intake at 6 months was significant at the *P* < .20 level in univariable analysis and if the factor was not known to be on the causal pathway between STH infection and milk intake. Only factors meeting these criteria were included in the final multivariable models to determine the most parsimonious models. STH prevalences and intensities were investigated by species and by intensity category (moderate‐and‐heavy vs. light‐and/or‐uninfected).

Multiple imputation by chained equations (MICE) with 20 imputations was used to impute infant water intake from human milk and nonmilk sources when outcome data for one of the two time points were missing. The MICE model included the following variables collected at baseline: infant sex, gestational age, maternal age, marital status, maternal education level, number of people residing in the household, SES index and intervention group. As one of the objectives of the study was to examine the presence of a differential response to the intervention by infant sex, the initial MICE model also included an interaction term for intervention group and infant sex. However, because no evidence of interaction was found, the interaction term was dropped from the final MICE model.

### 2.7 Ethical considerations

This study was conducted according to the guidelines laid down in the Declaration of Helsinki, and all procedures involving human subjects were approved by the Asociación Civil Impacta Salud y Educación (Peru, 0101‐2013‐CE), the Instituto Nacional de Salud (Peru, 12‐198/GEN/ACSA) and the McGill University Health Centre (Canada, 12‐198 GEN). Written informed consent was obtained from all mothers and fathers of participating infants for the participation of mother–infant pairs in the research study. If the mother was under the legal age of consent (i.e., 18 years), assent was obtained, and written informed consent was obtained by her legal guardian or spouse. Oversight for the parent trial was undertaken for the duration of the trial by an external Data Safety and Monitoring Committee. The trial entitled ‘Postpartum Deworming: Improving Breastfeeding and Optimizing Infant Growth’ is registered at Clinicaltrials.gov (NCT01748929).

## 3 RESULTS

### 3.1 Enrolment and study population

**FIGURE 1:**
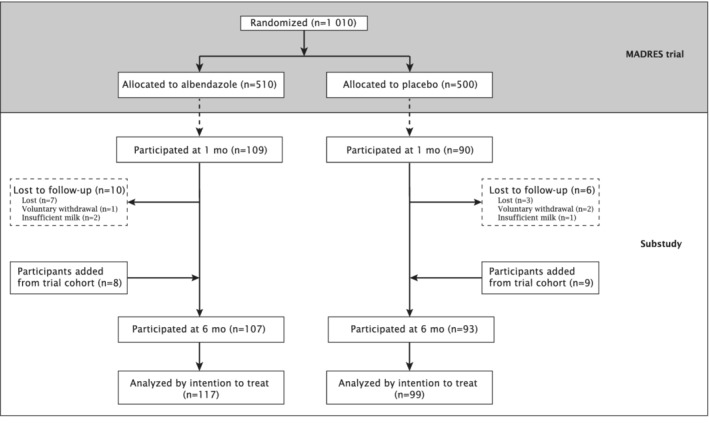
Participant flow throughout the Maternal Deworming Research Study (MADRES) trial and the milk intake study

A total of 1,010 mother–infant pairs were enrolled into the parent trial and randomized to receive albendazole (*n* = 510) or placebo (*n* = 500) between February and August 2014 (Mofid et al., 2017). Between April and December 2014, a total of 216 mother–infant pairs participated in the milk intake study (i.e., 199 pairs at 1 month and 200 pairs at 6 months) (Figure 1). Baseline characteristics of those who participated in the study, by intervention group, are summarized in Table 1. Overall, groups were similar; however, some differences were observed in maternal age and peri‐urbanicity of the home.

**TABLE 1:** Baseline socio‐demographic characteristics of mothers and infants who participated in the milk intake study, by intervention group, Iquitos, Peru (February–August 2014)

Characteristic	Albendazole		Placebo	
	(*n* = 117)^a^		(*n* = 99)^a^	
**Maternal characteristics**				
Age (years), (*SD*)	24.4	(6.7)	26.4	(7.8)
Married or cohabiting, *n* (%)	104	(88.9)	88	(88.9)
Primigravida, *n* (%)	32	(27.4)	23	(23.2)
Less than secondary school education, *n* (%)	72	(61.5)	68	(68.7)
Work outside the home, *n* (%)	11	(9.4)	3	(3.0)
Vaginal delivery, *n* (%)	99	(84.6)	78	(78.8)
**Infant characteristics**				
Male, *n* (%)	59	(50.4)	51	(51.5)
Birthweight (kg), (*SD*)	3.2	(0.4)	3.2	(0.4)
Birth length (cm), (*SD*)	48.7	(1.8)	48.7	(1.7)
Birth head circumference (cm), (*SD*)	33.7	(1.3)	33.7	(1.2)
Gestational age (weeks), (*SD*)	38.7	(0.8)	38.6	(1.0)
Apgar score at 5 min, (*SD*)	9.8	(0.5)	9.7	(0.6)
**Household characteristics**				
Peri‐urban or rural residence, *n* (%)	112	(95.7)	99	(100.0)
Access to potable water in home, *n* (%)	83	(70.9)	75	(75.8)
Home with dirt or wooden floor, *n* (%)	72	(61.5)	70	(70.7)
Number of people residing in household, (*SD*)	6.3	(2.8)	6.2	(2.6)

Abbreviation: *SD*, standard deviation.

^a^ Includes mother–infant pairs who participated in the study at 1‐ and/or 6‐month time point. Refer to Figure 1.

### 3.2 Follow‐up

Follow‐up for the 1‐month visit took place between April and August 2014 and follow‐up for the 6‐month visit between August and December 2014. Of the original 199 mother–infant pairs who participated at 1 month, 183 (92.0%) also completed the 6‐month assessment (Figure 1). The main reasons why those who completed the first assessment could not complete the second assessment were relocation to another city or temporarily away from the study area at the time of assessment (*n* = 10); withdrawal from the study (*n* = 3); and cessation of breastfeeding (*n* = 3). Participants who were lost to follow‐up were similar to their replacements in terms of maternal, child and household characteristics. Missing data occurred only due to loss‐to‐follow‐up; all baseline data, breastfeeding status and STH infection results were 100% complete. Infant milk intake was imputed for 15.3% (33/216) of the study.

At 1‐month postpartum, the prevalence of reported exclusive breastfeeding, and combined exclusive and predominant breastfeeding, was 48.2% (95% CI: 41.3%, 55.2%) and 92.5% (95% CI: 87.8%, 95.4%), respectively. At 6‐month postpartum, the prevalence of reported exclusive breastfeeding, and combined exclusive and predominant breastfeeding, was 1.0% (95% CI: 0.2%, 4.0%) and 10.0% (95% CI: 6.5%, 15.0%), respectively. There were no statistically significant differences between intervention groups with respect to reported breastfeeding status at either time point.

### 3.3 Effect of maternal postpartum deworming on infant milk intake

Mean infant milk intake at 1‐month postpartum was 756 ± 16 and 774 ± 18 mL day^−1^ in the albendazole and placebo groups, respectively (Table 2). Mean infant milk intake at 6‐month postpartum was 903 ± 16 and 908 ± 18 mL day^−1^ in the albendazole and placebo groups, respectively. There were no statistically significant differences in mean infant milk intake between intervention groups at 1‐ or 6‐month postpartum. Similar findings were obtained from complete‐case and per‐protocol analyses and in subgroup analyses restricted to mothers who reported exclusive breastfeeding (data not shown).

**TABLE 2:** Impact of maternal postpartum deworming on infant milk intake at 1‐ and 6‐months postpartum, Iquitos, Peru (April–December 2014)

Outcome	Albendazole		Placebo	
	*n* = 117^a^		*n* = 99^a^	
**1 month**				
Milk intake (mL day^−1^), mean (SE)	756	(16)	774	(18)
Unadjusted difference (95% CI)	−18	(−65, 30)	*Reference*	
*P value*	0.471			
Adjusted^b^ difference (95% CI)	−18	(−66, 30)	*Reference*	
*P value*	0.458			
**6 months**				
Milk intake (mL day^−1^), mean (SE)	903	(16)	908	(18)
Unadjusted difference (95% CI)	−5	(−52, 43)	*Reference*	
*P value*	0.849			
Adjusted^b^ difference (95% CI)	−11	(−58, 36)	*Reference*	
*P value*	0.657			

Abbreviations: CI, confidence interval; SE, standard error.

^a^ ITT regression analysis includes mother–infant pairs who participated in the study at 1‐ and/or 6‐month time point. Refer to Figure 1.

^b^ Adjusted for maternal age, education, SES index, gestational age and infant sex.

### 3.4 Maternal STH infection status and infant milk intake

At 6‐month postpartum, 200 mothers provided a stool specimen for evaluation of STH infection status. The prevalence and intensity of infection with any one of the STH species, as well as species‐specific infection, by intervention group, are presented in Table 3. The prevalence of any STH infection was estimated to be 26.2% in the albendazole group and 48.4% in the placebo group, with the prevalences of *A. lumbricoides* and *T. trichiura* infections exceeding 30% in those who received placebo. Co‐infections occurred in 12.5% (*n* = 25) of the study population, with most co‐infections reported in the placebo group (i.e., six co‐infections in the albendazole group vs. 19 co‐infections in the placebo group). The most frequent co‐infection was that of *A. lumbricoides* with *T. trichiura*.

**TABLE 3:** Soil‐transmitted helminth species‐specific prevalence, prevalence of moderate‐and‐heavy intensity (MHI) infection and mean intensity at 6‐months postpartum, by intervention group, Iquitos, Peru (August–December 2014)

Species	Albendazole		Placebo	
	*n* = 107^a^		*n* = 93^a^	
***Ascaris lumbricoides***				
Prevalence % (95% CI)	11.2	(6.4, 18.8)	32.3	(23.5, 42.5)***
Prevalence of MHI % (95% CI)	2.8	(0.9, 8.4)	8.6	(4.3, 16.4)
Intensity (mean epg^b^) (95% CI)	2143.6	(815.2, 5636.5)	2998.8	(1833.8, 4903.9)
***Trichuris trichiura***				
Prevalence % (95% CI)	20.6	(13.9, 29.4)	32.3	(23.5, 42.5)
Prevalence of MHI % (95% CI)	0.9	(0.1, 6.5)	2.2	(0.5, 8.3)
Intensity (mean epg^b^) (95% CI)	172.0	(122.9, 240.8)	184.5	(135.3, 251.7)
**Hookworm**				
Prevalence % (95% CI)	0.9	(0.1, 6.5)	7.5	(3.6, 15.1)*
Prevalence of MHI % (95% CI)^c^	0		0	
Intensity (mean epg^b^) (95% CI)	N/A^d^		82.8	(37.6, 182.3)
**Any STH**				
Prevalence % (95% CI)	26.2	(18.6, 35.4)	48.4	(38.3, 58.6)**

Abbreviations: CI, confidence interval; epg, eggs per gram; MHI, moderate‐and‐heavy intensity.

^a^ Includes mothers who provided a stool specimen at the 6‐month visit (107 in the albendazole group and 93 in the placebo group).

^b^ Geometric mean among infected only.

^c^ At the 6‐month time point, no participants were infected with moderate‐and‐heavy hookworm infection.

^d^ Mean epg cannot be calculated because there was only a single hookworm‐positive specimen in the albendazole group.

Asterisks highlight significant P‐values:

* < 0.05.

** < 0.01.

*** < 0.001.

Infants of mothers infected with *T. trichiura* (*n* = 52) had higher mean milk intakes than infants of mothers who were not infected with *T. trichiura* (*n* = 148) (adjusted mean difference: 70 mL day^−1^; 95% CI: 20, 120) (Table 4). No statistically significant difference in mean infant milk intake was observed among infants of mothers with any moderate‐and‐heavy species‐specific infection intensity compared with the species‐specific combined comparative group of those with light intensity infection and those uninfected (Table 5). Additionally, there was no statistically significant difference in mean infant milk intake when comparing infants of mothers with any species‐specific moderate‐and‐heavy intensity infection to infants of mothers who were uninfected (data not shown).

**TABLE 4:** Association between prevalence of soil‐transmitted helminth infection and infant milk intake at 6‐months postpartum, Iquitos, Peru (August–December 2014)

Outcome	Infected^a^		Uninfected^a^	
***Ascaris lumbricoides***				
*n*	42		158	
Mean milk intake (*SD*) (mL day^−1^), 6 months	880	(199)	909	(153)
Unadjusted difference (95% CI)	−29	(−85, 27)	*Reference*	
*P value*	0.309			
Adjusted^b^ difference (95% CI)	−36	(−93, 22)	*Reference*	
*P value*	0.222			
***Trichuris trichiura***				
*n*	52		148	
Mean milk intake (*SD*) (mL day^−1^), 6 months	960	(163)	884	(160)
Unadjusted difference (95% CI)	76	(25, 127)	*Reference*	
*P value*	0.004**			
Adjusted^b^ difference (95% CI)	70	(20, 120)	*Reference*	
*P value*	0.007**			
**Hookworm**				
*n*	8		192	
Mean milk intake (*SD*) (mL day^−1^), 6 months	852	(114)	905	(165)
Unadjusted difference (95% CI)	−53	(−170, 63)	*Reference*	
*P value*	0.370			
Adjusted^b^ difference (95% CI)	−35	(−150, 80)	*Reference*	
*P value*	0.550			
**Any STH**				
*n*	73		127	
Mean milk intake (*SD*) (mL day^−1^), 6 months	922	(186)	892	(149)
Unadjusted difference (95% CI)	30	(−17, 77)	*Reference*	
*P value*	0.210			
Adjusted^b^ difference (95% CI)	19	(−30, 68)	*Reference*	
*P value*	0.442			

Abbreviations: CI, confidence interval; *SD*, standard deviation.

^a^ Includes mothers who provided a stool specimen at the 6‐month visit (107 in the albendazole group and 93 in the placebo group).

^b^ Adjusted for intervention group, maternal age, maternal employment status, parity, SES index, mode of delivery and infant sex.

Asterisks highlight significant P‐values

* < 0.05.

** < 0.01.

*** < 0.001.

**TABLE 5:** Association between intensity of soil‐transmitted helminth infection and infant milk intake at 6‐months postpartum, Iquitos, Peru (August–December 2014)

Outcome^a^	Moderate‐and‐heavy infection^b^		Light‐and‐uninfected^b^	
***Ascaris lumbricoides***				
*n*	11		189	
Mean milk intake (*SD*) (mL day^−1^), 6 months	907	(243)	903	(159)
Unadjusted difference (95% CI)	4	(−97, 104)	*Reference*	
*P value*	0.941			
Adjusted^c^ difference (95% CI)	−5	(−103, 93)	*Reference*	
*P value*	0.925			
***Trichuris trichiura***				
*n*	3		197	
Mean milk intake (*SD*) (mL day^−1^), 6 months	810	(313)	905	(161)
Unadjusted difference (95% CI)	−95	(−282, 93)	*Reference*	
*P value*	0.321			
Adjusted^c^ difference (95% CI)	−70	(−254, 113)	*Reference*	
*P value*	0.450			
**Any STH**				
*n*	12		188	
Mean milk intake (*SD*) (mL day^−1^), 6 months	917	(234)	902	(159)
Unadjusted difference (95% CI)	15	(−81, 111)	*Reference*	
*P value*	0.758			
Adjusted^c^ difference (95% CI)	13	(−81, 107)	*Reference*	
*P value*	0.791			

Abbreviations: CI, confidence interval; *SD*, standard deviation.

^a^ At the 6‐month time point, no participants were infected with moderate‐and‐heavy hookworm infection.

^b^ Includes mothers who provided a stool specimen at the 6‐month visit (107 in the albendazole group and 93 in the placebo group).

^c^ Adjusted for intervention group, maternal age, maternal employment status, parity, SES index, mode of delivery and infant sex.

## 4 DISCUSSION

This study is the first to evaluate the impact of maternal postpartum deworming and STH infection on infant milk intake in a human population. Previous research has been undertaken on the impact of gastrointestinal nematode infections and deworming on milk production in ruminants (Charlier et al., 2014). Reviews of the veterinary literature have found that deworming increased ruminant milk production from between 0.35 and 1 kg cow^−1^ per day (Charlier et al., 2009; Sanchez et al., 2004). Therefore, it might be expected that, by reducing maternal STH infection burden in the early postpartum period (with a single dose of albendazole), infant milk intake over the first 6 months of life would be improved.

Infant milk intake observed in our study was within the WHO reference ranges for 1‐ and 6‐month‐old exclusively breastfed infants (Butte et al., 2002). Several hypotheses may be advanced to explain the similar levels of mean infant milk intake in the two intervention groups at both 1‐ and 6‐month postpartum: (1) The prevalence of STH infection would be expected to be similar in both groups at baseline and has similar effects on colostrum and early milk available to the infant; (2) the prevalence of STH infection and the prevalence of each STH species might have been too low to observe an effect (i.e., the uninfected proportion within each intervention group has a dilution effect on measuring the effect of deworming); (3) there might not have been enough time to observe the positive effects of deworming with respect to the biological mechanisms involved in the production of breast milk by the 1‐month time point; and (4) there was sufficient time for mothers in the albendazole group to be reinfected before the 6‐month time point, thereby reducing STH prevalence between the intervention groups.

The finding that infants of mothers infected with *T. trichiura* had higher milk intakes than infants of mothers who were not infected with *T. trichiura* is of interest. Although there is the possibility that it is a spurious result, it may also be that some biological pathway involving a host‐pathogen response to *T. trichiura* infection, especially if that infection is chronic, is ultimately manifested in higher infant milk intakes. Compared with other STH infections like hookworm, *T. trichiura* infection is notoriously difficult to treat with albendazole (mean efficacy of 30.7% [21.0%, 42.5%]) in large‐scale deworming programmes, resulting in a more chronic infection (Moser et al., 2017). *T. trichiura* infection (in addition to hookworm infection) has been shown to be associated with anaemia in pregnant women (Gyorkos et al., 2011, 2012), which is likely to influence breastfeeding. How maternal *T. trichiura* infection impacts nutrient transfer or milk intakes of infants remains to be understood, but evidence from nutrient supplementation trials, including iron supplementation, clearly documents benefits in terms of both maternal and infant health outcomes (Christian et al., 2015; Dror & Allen, 2018).

Our study had several limitations. Baseline assessment of STH infection in the trial population was not possible to ascertain because of ethical considerations in withholding treatment among women who would have been found to be positive. For this reason, STH prevalence was only ascertained at the 6‐month time point of follow‐up after which albendazole was administered to all mothers in both groups. As a result, it was not possible to identify the infection status of individual women prior to treatment allocation. However, the assumption would be that the prevalence observed in the placebo group would reflect the prevalence in the entire study population at baseline. The sample size of 200 may not have been sufficient to observe a difference, given that the sample size was estimated on the basis of observing a statistical difference in milk intake and did not take into consideration the prevalence of STH infection in the study population. A larger sample size might have been able to uncover smaller STH‐attributable effects on milk intake. Some data were missing, although this was minimal; missing outcome data were imputed, and missingness was not expected to have influenced the overall results. Lastly, the proportion of mothers who exclusively breastfed their infants was extremely low, especially at the 6‐month timepoint, reflecting cultural practices of introducing especially liquids before 6 months of age.

There are important strengths of this study. There was added value of undertaking a milk intake study with a relatively large sample size (i.e., 200 mother–infant pairs) within a large randomized controlled trial design, with only a small loss to follow‐up over the 6 months of the study. The collection of urine from both mother and infant in their home was standardized and rigorously performed. The use of the deuterium oxide technique for measuring infant milk intake is considered the gold standard by which the turnover rate of body water is calculated following oral ingestion of a stable isotope tracer by the lactating mother (Coward et al., 1982). It is ideal to use in field settings because it is precise, requires minimal time commitment and does not interfere with the normal mode of feeding of the mother–infant pair (International Atomic Energy Agency, 2010).

In this study, postpartum deworming (i.e., a single 400‐mg dose of albendazole administered prior to hospital discharge after delivery) did not affect infant milk intake in the first 6 months of life. The observation that infants of *T. trichiura*‐infected mother had higher milk intakes merits further investigation. As deworming programmes will increasingly include women of reproductive age (from adolescent to adult and, in the pregnant woman, from antenatal to postpartum care), future research will be helpful in documenting their impact on both mother and child (World Health Organization, 2017, 2018). It would be important to also investigate short‐ and longer‐term effects, compliance levels and programme costs. Evidence originating in endemic settings of known higher STH prevalence and moderate and heavy levels of STH intensity would be ideal.

Offering deworming medication to new mothers before they leave the hospital will not only reduce STH‐related morbidity in the women themselves, but it is likely to also result in cost savings to the health system. Together with a school‐based deworming programme, such a health system‐based programme offered to women would provide deworming to all recognized population groups at highest risk of STH‐related morbidity (World Health Organization, 2017). Such an approach might well accelerate progress towards achieving the goal of STH elimination by 2030.

## CONFLICTS OF INTEREST

The authors declare that they have no conflicts of interest.

## CONTRIBUTIONS

LSM, BB and TWG were responsible for conception of the research question and protocol development, with critical input from MC, AM, ER, GSM, JV and LHA. LSM, LP and HR were responsible for on‐site trial management including training, logistics, development of study tools, coordination of samples and overseeing of data collection. LSM, TWG and ER were responsible for the data analysis. LSM and TWG were responsible for manuscript preparation. All authors contributed to the interpretation of data and the preparation and revision of the manuscript.

## Data Availability

The data that support the findings of this study are available from the corresponding author upon reasonable request.
